# Critical Song Features for Auditory Pattern Recognition in Crickets

**DOI:** 10.1371/journal.pone.0055349

**Published:** 2013-02-20

**Authors:** Gundula Meckenhäuser, R. Matthias Hennig, Martin P. Nawrot

**Affiliations:** 1 Theoretical Neuroscience and Neuroinformatics, Institute of Biology, Freie Universität Berlin, Berlin, Germany; 2 Department of Biology, Humboldt-Universität zu Berlin, Berlin, Germany; 3 Theoretical Neuroscience and Neuroinformatics, Institute of Biology, Freie Universität Berlin, Berlin, Germany; University of Western Australia, Australia

## Abstract

Many different invertebrate and vertebrate species use acoustic communication for pair formation. In the cricket *Gryllus bimaculatus*, females recognize their species-specific calling song and localize singing males by positive phonotaxis. The song pattern of males has a clear structure consisting of brief and regular pulses that are grouped into repetitive chirps. Information is thus present on a short and a long time scale. Here, we ask which structural features of the song critically determine the phonotactic performance. To this end we employed artificial neural networks to analyze a large body of behavioral data that measured females’ phonotactic behavior under systematic variation of artificially generated song patterns. In a first step we used four non-redundant descriptive temporal features to predict the female response. The model prediction showed a high correlation with the experimental results. We used this behavioral model to explore the integration of the two different time scales. Our result suggested that only an attractive pulse structure in combination with an attractive chirp structure reliably induced phonotactic behavior to signals. In a further step we investigated all feature sets, each one consisting of a different combination of eight proposed temporal features. We identified feature sets of size two, three, and four that achieve highest prediction power by using the pulse period from the short time scale plus additional information from the long time scale.

## Introduction

**Figure 1:**
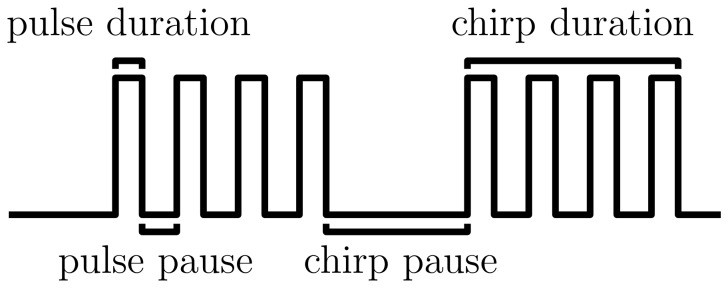
Artificial song pattern of the cricket *Gryllus bimaculatus* and its temporal features. Typically, a calling song consists of repetitive pulses that are grouped into chirps. The temporal structure of an artificial song pattern is fully determined by four descriptors, e.g. the duration and pause for both pulses and chirps. Four additional descriptors are frequently used to characterize cricket songs, namely the period (the sum of duration and pause), and the duty cycle (the ratio of duration and period) for both, the short and the long time scale.

Acoustic communication plays a key role for mating behavior in many different species, most prominently in birds [1], fish [2], amphibians [3], and insects [4]–[6]. In the cricket species *Gryllus bimaculatus* males produce calling songs by rubbing their wings and females use these songs to localize the potential partner. If females recognize the conspecific song and rate it as attractive they approach the singing male, a behavior called phonotaxis (for an overview see [4] and [7]). The natural pattern of a calling song consists of repetitive pulses that are grouped into pulse trains called chirps [8]. The attractiveness of different patterns can be easily tested under laboratory conditions by monitoring the phonotactic behavior of females toward artificial signals [9], [10]. Extensive phonotaxis experiments suggested that the brain processes the patterns in the temporal domain [9], [11] rather than in the spectral domain as has been proposed earlier [12]. Schneider and Hennig [13] provided evidence that females evaluate only the coarse temporal structure of a pattern. Consequently, the abstract song pattern of *Gryllus bimaculatus* can be described with four independent parameters [14], for example the pulse duration, pulse pause, chirp duration, and chirp pause (see Figure 1). However, it is not clear whether these four cues are analyzed independently in the cricket brain. The period, that is the sum of duration and pause, as well as the duty cycle, that is the ratio of duration and period, for both pulses and chirps have also been implicated as relevant descriptors [9], [11], [14]. Behavioral experiments [9] show that a pulse period of 40 ms at a pulse duty cycle of 0.5 elicits highest phonotactic scores. For the organization of the chirps Grobe et al. [11] observed optimal ranges between 200 and 500 ms for the chirp period, provided that the chirp duty cycle lies between 0.3 and 0.7. However, the relative importance of each of these song features is as yet unclear.

Here, we employ artificial neural networks, which are also known as multilayer perceptrons, to analyze a large body of behavioral data obtained in phonotaxis experiments. We provide a detailed investigation of the relevance of individual song parameters on a quantitative measure that rates phonotactic behavior. Our models provide quantitative predictions for the attractiveness of hitherto untested song parameters, which helps guiding future phonotaxis experiments. Finally, we carefully interpret our results with respect to the underlying neural processing employed for acoustic pattern evaluation in the cricket brain.

## Materials and Methods

### Behavioral Experiments and Data

We used behavioral tests to measure the phonotactic score of the cricket *Gryllus bimaculatus* as explained in detail in [9]. In brief, female crickets were placed on top of a trackball system that records their 2D walking trace. The females were presented with song patterns that mimic natural calling songs. These were constructed by amplitude modulated sinusoidal signals with a carrier frequency of 4.5 kHz. The amplitude was modulated to construct a periodical series of rectangular sound pulses that are grouped into chirps (see Figure 1). As a measure for the attractiveness of a particular song pattern, we computed the phonotactic score according to the formula in Schul [15]. The phonotactic score is an integral measure that involves the walking length, the accuracy of the course maintenance, and the orientation of the female. It assumes values between −1 and 1, whereat a value close to one indicates a high level of attractiveness of the tested song pattern. For this study, we grouped data from experiments of 218 song patterns differing in their temporal parameters each of which was presented to several female crickets (mean: 31, range: 8–225). For each song the phonotactic score was averaged across individual animals.

The data set was preprocessed as follows. First, we examined the distribution of the response values of the song patterns: 35% of the patterns were unattractive with a phonotactic score smaller than 0.2, 48% were intermediate between 0.2 and 0.6, and 17% were attractive with a value greater than 0.6 [9], [11]. Then we split the data set into a training data set and a test data set of 200 and 18 data points, stratified according to the above allocation of unattractive, intermediate and attractive songs. This method is known as stratified sampling and was applied whenever data sets were divided into subsets. Then, we whitened the temporal calling song features of the training data set and applied the obtained transformation to the features of the test data set as well. In the whitening process, the features are first projected onto their principal components which removes linear correlations across features and then each feature is normalized to zero mean and unit variance. This linear coordinate transformation is widely used to preprocess the data before applying regression methods such as artificial neural networks [16].

### Model

#### Artificial neural networks

**Figure 2:**
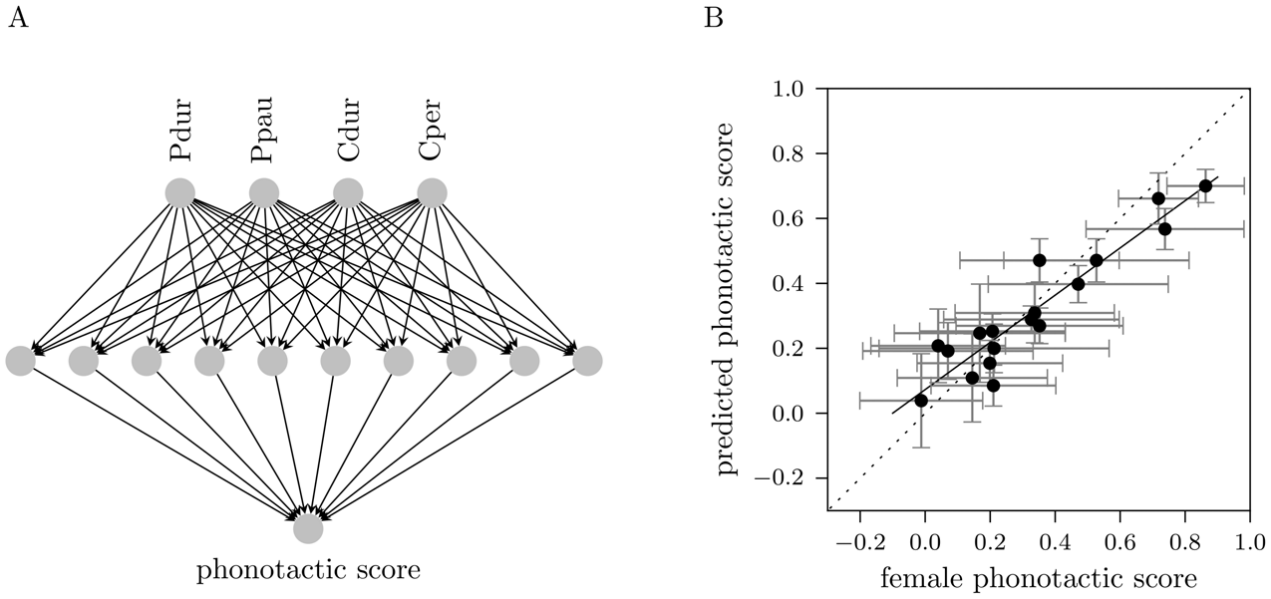
Network diagram and predictive performance of the best 4-feature model. (A) The network diagram consists of four input neurons representing temporal calling song features, which project to input-evaluating neurons in the hidden layer. These in turn project to the output neuron mimicking the relative phonotactic score; abbreviations: Pdur - pulse duration, Ppau - pulse pause, Cdur - chirp duration, Cper - chirp period. (B) Correlation between the phonotactic score of 18 test samples predicted by the best 4-feature model and the experimentally measured scores. Each dot shows the mean phonotactic score for a given song pattern that was presented to on average 31 females and tested for 100 times with the model. The errorbars indicate standard deviation across individual females (horizontal) and across 100 repeated model simulations (vertical). The solid regression line has a slope of 0.73. The performance: 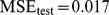 and 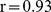.

Artificial neural networks are commonly employed for regression tasks [16], that is in our case to predict the phonotactic score from untested patterns. Figure 2A shows an example of a network diagram with four input variables that represent the features of a calling song, ten neurons in the hidden layer and one output neuron that represents the corresponding phonotactic score. In detail, the information about the features is forward propagated as follows: input variables 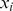 that represent calling song features are linearly combined to activations 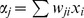 of hidden neuron 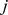, where 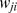 denotes the synaptic weight between input neuron *i* and hidden neuron *j*. Then, the activations of each hidden neuron are transformed with a nonlinear sigmoidal function 
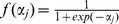
. Finally, the output variable 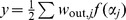 is computed, where 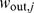 indicates the synaptic weight between hidden neuron *j* and the output neuron. Thus, in artificial neural networks the temporal calling song features are nonlinearly processed to predict the phonotactic score. We implemented artificial neural networks in the Python programming language, using the Fast Artificial Neural Network Library [17].

#### Training and validation

For training the synaptic weights, we chose the RProp algorithm which is a well-established supervised learning technique for multilayer feed-forward networks [18]. The algorithm uses a training data set to update the randomly initialized weights in each training cycle such that the mean squared error between the model’s prediction and the experimentally observed phonotactic score is minimized. We used the whitened training data set to perform a stratified 5-fold cross validation for training and validating networks. The training was stopped after 10,000 cycles. This stopping criterion enabled us to compare the performance of networks with different architectures. To produce a single error estimation, the mean squared errors for validation (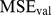) and training (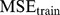) were averaged over folds. In order to account for random initialization of the weights, the 5-fold cross validation was repeated for 100 times and we calculated the mean and standard deviation of the 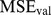 and 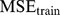.

#### Model selection

In a first step we determined the appropriate number of neurons in the hidden layer by comparing the validation errors of networks with 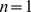 to 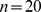 hidden neurons. In detail, for each 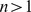, we calculated the percent change of the validation error with respect to the network consisting of 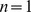 neuron. Then, we chose the smallest *n* such that networks with 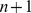 hidden neurons lead to an improved performance of no more than 1% as compared to networks with *n* neurons. This criterion ensured to select a network with high predictive power on the one hand and a simple model architecture on the other hand.

#### Performance

To obtain an unbiased estimate of a network’s ability to generalize we used the test data set of 18 song patterns to test the network’s performance. Therefore, we trained a network with the whitened training data for 10,000 cycles and ran it with the test data set. Again, we repeated this for 100 times and averaged the network’s prediction. Then we calculated the mean squared test error (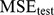) as well as the linear Pearson correlation coefficient between the averaged network’s predictions and the mean phonotactic scores averaged over females.

#### Prediction

To predict the phonotactic score of an untested song pattern, we first trained a chosen network over 10,000 cycles with whitened features and the corresponding phonotactic score of the initial feature set of 218 data points. Then, we transformed the features of the untested song pattern with the transformation obtained in the whitening process of the features belonging to the initial data set. Next, we run the trained model with the transformed features of the untested song pattern. Finally, we repeated this training and prediction procedure for 100 times and averaged the phonotactic scores across the repetitions.

#### Feature selection

We considered in total eight different temporal features of a song pattern that have been previously used as descriptors. This is a redundant set of descriptors as four features, two on the short and two on the long time scale, are sufficient to fully define the song pattern. However, it is not a priori known, which set of features will best describe the behavioral data. Thus, we investigated all 255 feature sets, each one consisting of a different combination of the eight temporal features. For each feature set, we trained and validated models for a different number of hidden neurons followed by the selection of the appropriate model, as described above.

## Results

Our analyses comprised a large body of behavioral data from experiments in which artificial calling songs were presented to female crickets under systematic variation of the song parameters. The phonotactic behavior was monitored with a single quantity, the phonotactic score. The acoustic pattern of an artificial song is shown in Figure 1. We trained artificial neural networks that receive as input the values of a particular set of song features to predict the phonotactic score. First, we considered feature sets made up by two features on the short pulse time scale and two on the long chirp time scale and analyzed how well an artificial neural network trained on parts of the experimental data can predict the phonotactic score on the remaining test data. In order to investigate the interplay of pulse and chirp information with respect to the phonotactic score we systematically varied pulse period and chirp period. Finally, we compared feature sets, each one consisting of a different combination of temporal features, in order to determine those features that are most efficient in correctly predicting average phonotactic behavior.

### Predictive Performance of Models Using Full Temporal Pattern Information

How well can we predict the behavioral outcome in an experimental trial based on the particular song pattern that was presented? To answer this question we trained and validated different artificial neural networks on non-redundant input features using a cross-validation procedure. From a total of eight potential features we investigated all combinations made up by two features on the short pulse time scale and two on the long chirp time scale that together fully determine the temporal song structure (see Figure 1). The best performing 4-feature model was selected based on the validation error. It used pulse duration, pulse pause, chirp duration, and chirp period as input features and comprised 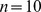 hidden neurons. The network diagram is shown in Figure 2A.

The average performance of this 4-feature model was quantified on the test data set as shown in Figure 2B, where each point corresponds to one song pattern and the model prediction is plotted against the average phonotactic score computed from the animals’ behavior. The predicted response values for the test data set were highly correlated with the experimentally measured responses, that is with a linear correlation coefficient of 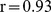. The mean squared error between the predictions and the experimental measurements was 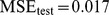. The vertical errorbars indicate standard deviation, indicating the prediction variability of the best 4-feature model that was simulated for 100 times toward the same calling song. The main source for this variability is that before training the weights were initialized randomly, which resulted in slightly different predictions for one song pattern. The horizontal errorbars indicate inter-individual response variability of different females toward the same song.

### Fusion of the Short and Long Time Scale

**Figure 3:**
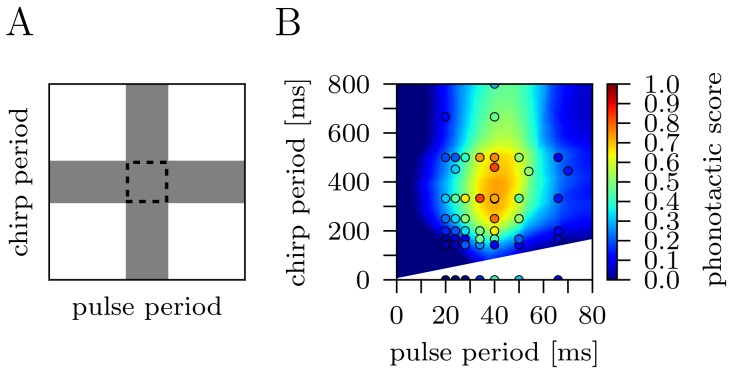
Interaction of the short and long time scale. (A) Sketch of a logical AND-operation (central square) and an OR-operation (gray shading). (B) Chirp period - pulse period response field predicted by the best 4-feature model (pulse duration, pulse pause, chirp duration, chirp period). The dominant circular area of highest response values suggests an AND-operation. Circles indicate experimentally measured phonotactic scores.

Female crickets use information from both, the pulse pattern and the chirp pattern to recognize and evaluate the conspecific song. How is this information on the short pulse and the long chirp time scales combined by female crickets during auditory processing? We hypothesize two basic models as sketched in Figure 3A: in case of a logical AND-operation only an attractive pulse structure in combination with an attractive chirp structure generates highest phonotactic scores. This would indicate a synergistic processing. In contrast, a logical OR-operation requires either a suitable pulse or an attractive chirp structure to drive high phonotactic scores, thus optimal parameters for both time scales do not transmit extra information. The latter behavior is known as hypo-additive effect [19]. We evaluated the best 4-feature model (pulse duration, pulse pause, chirp duration, and chirp period) for different patterns by systematic variation of chirp and pulse periods. While varying the periods we fixed the duty cycles at 0.5, which ensured that one parameter of each time scale was in an attractive range [9], [11]. The plane spanned by the chirp period and the pulse period in Figure 3B shows highest response values for patterns with a chirp period between 250 and 500 ms and pulse periods from 35 to 45 ms. The maximal response value was obtained for a pattern with a pulse period of 40 ms and a chirp period of 340 ms. The dominant circular shape of highest responses suggested that the model approximates a logical AND-operation for high phonotactic scores.

### Selection of the Most Informative Song Features

**Figure 4:**
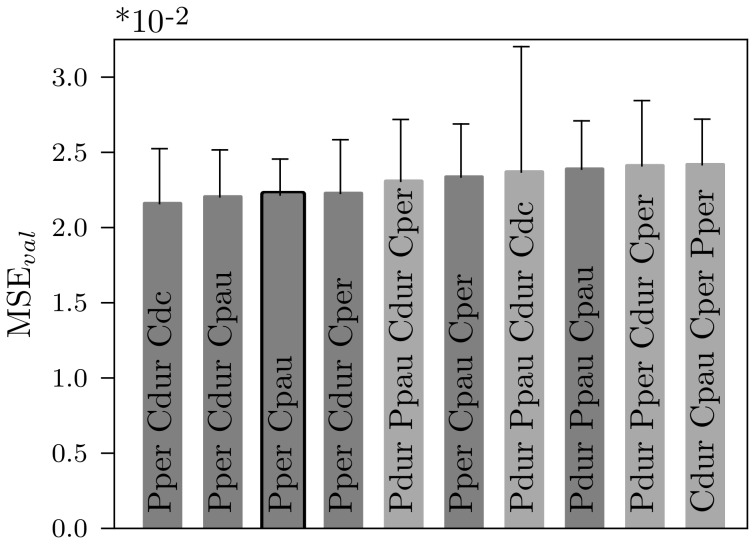
Ten best performing models. Model of size four (light gray), three (dark gray), and two (black edging) are ranked top ten. The overall best performing model uses the pulse period, chirp duration, and chirp duty cycle. The best 2-feature model (pulse period and chirp pause) did not perform significantly different (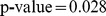 for a two-sided Wilcoxon rank-sums test; significance level of 0.01). The best 4-feature model (pulse duration, pulse pause, chirp duration, and chirp period) performed significantly worse than the best 3-feature model (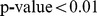 for a two-sided Wilcoxon rank-sums test). Abbreviations: Pdur - pulse duration, Ppau - pulse pause, Pper - pulse period, Pdc - pulse duty cycle, Cdur - chirp duration, Cpau - chirp pause, Cper - chirp period, Cdc - chirp duty cycle. The models were validated 100 times and errorbars indicate standard deviation.

Which are the critical temporal song features that carry the most information for phonotaxis? A number of different song parameters have previously been tested experimentally and several have been suggested to be of particular importance. We considered a total of eight temporal features, namely duration, pause, period, and duty cycle for both pulses and chirps, as introduced in Figure 1. Above we already presented a model that uses two features from the short time scale (pulse duration and pulse pause) and two features from the long time scale (chirp duration and chirp period). However, it is not clear, which set of features will best describe the behavioral data. Thus, we investigated all possible feature sets, each one consisting of a different combination of the eight temporal features and compared the prediction accuracy of the corresponding models. Figure 4 shows the ten best models. The overall best performance with respect to the validation error was obtained for the 3-feature model that uses pulse period, chirp duration, and chirp duty cycle as input and 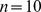 neurons in the hidden layer. All models using the pulse period plus two features from the long time scale were among the ten best performing networks that use three features as input. Surprisingly, the best 2-feature model that only uses pulse period and chirp pause and 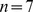 hidden neurons did not perform significantly different from the best 3-feature model (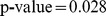 for a two-sided Wilcoxon rank-sums test; significance level of 0.01). The feature combinations of pulse period plus one chirp feature are the four best in the class of models that only use two features as input. In contrast, the best 4-feature model that uses pulse duration, pulse pause, chirp duration, and chirp period as input and 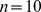 hidden neurons performed significantly worse than the best 3-feature model (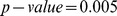 for a two-sided Wilcoxon rank-sums test; significance level of 0.01). Models with only one or more than four features as input were not ranked top ten.

### Model Predictions for Pulse and Chirp Response Fields

**Figure 5:**
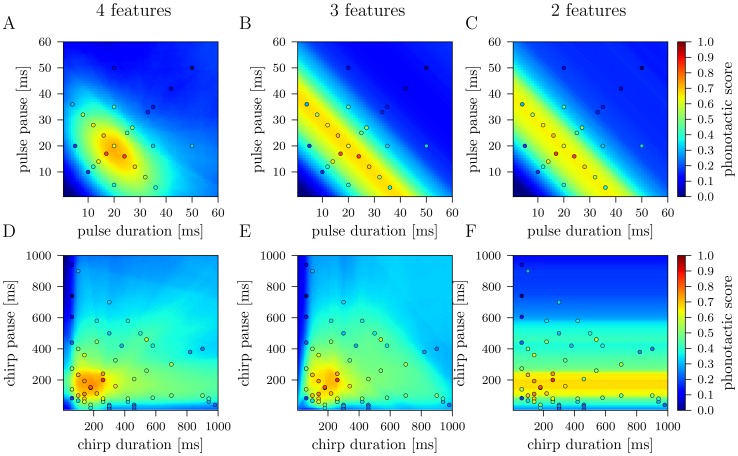
Pulse and chirp response fields predicted by the best 4-feature, 3-feature and 2-feature model. (A) The pulse response field of the best 4-feature model shows highest phonotactic scores for patterns that are accumulated in an oval bounded by pulse periods of 30 and 45 ms and pulse duty cycles of 0.4 and 0.7. The pulse response fields of the best 3-feature model (B) and the best 2-feature model (C) are clearly independent of the pulse duty cycle and show an extension on the diagonal defined by a pulse period of 40 ms. The chirp response field of the best 4-feature model (D) and the best 3-feature model (E) are qualitatively similar and reveal best scores for patterns with chirp durations and pauses between 200 and 300 ms. The best 2-feature model predicts highest scores for patterns with a chirp pause between 100 and 250 ms, irrespective of the chirp duration (F). Circles indicate experimentally measured phonotactic scores.

We investigated pulse and chirp response fields predicted by the best 4-feature, 3-feature, and 2-feature models. Pulse response fields describe two-dimensional subspaces spanned by the pulse duration and pulse pause of the eight dimensional feature space in which the attractiveness is color coded. To this end, we trained the models using all data of 218 songs and their phonotactic scores. For the best 4-feature model, we predicted the phonotactic scores for patterns with different pulse durations and pulse pauses but with a fixed chirp duration of 200 ms and a fixed chirp period of 333 ms that construct an attractive chirp structure [9]. The pulse response field of this model, as shown in Figure 5A, reveals an oval structure: song patterns with high phonotactic scores are displayed in an area bounded by pulse periods of 30 and 45 ms and pulse duty cycles of 0.4 and 0.7. For the best 3-feature and best 2-feature model we predicted responses toward patterns with different pulse periods but with a fixed chirp duration of 200 ms and a chirp duty cycle of 0.6 (best 3-feature model), and a fixed chirp pause of 133 ms (best 2-feature model). The pulse response fields of the best 3-feature model (Figure 5B) and the best 2-feature model (Figure 5C) were highly similar: due to the fact that only a single parameter on the short time scale was used we obtained a 1-dimensional structure where the phonotactic score varied along the diagonal defined by the pulse period. Particularly, the phonotactic scores were invariant under different pulse duty cycles. Higher phonotactic scores were in the range of 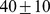 ms pulse period, which was consistent with predictions of the best 4-feature model.

Next, we analyzed the chirp response fields. We predicted the response values for song patterns with different chirp durations and chirp periods but a fixed pulse duration of 20 ms and a fixed pulse pause of 20 ms for the best 4-feature model. In case of the 3-feature model we predicted responses toward patterns with different chirp durations and chirp duty cycles but a fixed pulse period of 40 ms. The response fields of the best 4-feature (Figure 5D) and 3-feature model (Figure 5E) revealed highest phonotactic scores for chirp durations and pauses between 100 and 300 ms. The chirp response field of the 2-feature model, obtained by varying the chirp pause at a fixed pulse period of 40 ms, revealed a 1-dimensional structure in which the scores only vary for different chirp pauses, irrespective of the chirp duration, see Figure 5F. Here, highest phonotactic scores were predicted for chirp pauses between 100 and 250 ms.

## Discussion

In this study we trained artificial neural networks to predict the attractiveness of calling songs of the cricket *Gryllus bimaculatus*. We studied the dependence of the model performance on the parameters of the calling song and aimed to identify minimal subsets of temporal features that carried sufficient information to describe the experimentally measured behavioral performance.

### The most Relevant Song Features for Behavior

A number of different song parameters, namely the duration, pause, period, and duty cycle for both pulses and chirps are commonly used in cricket studies [9], [11], [12], [14], [20]. But, this set is overcomplete in the following sense: four features, two from each time scale, are sufficient to describe the artificial calling song. Thus, we investigated the performance of in total 255 models each one using a different set of song features. We identified three feature sets of different sizes that are best describing the behavioral data. The best 4-feature model, which used pulse duration, pulse pause, chirp duration and chirp period was ranked top ten (see Figure 4). The overall best model uses three features, the pulse period, chirp duration, and chirp duty cycle. Remarkably, the six combinations consisting of the pulse period plus two features from the long time scale are among the ten best performing sets of three features. Also the best 2-feature model uses the pulse period from the short time scale plus the chirp pause as input and regarding only models with two input features, the pulse period plus one feature from the long chirp time scale are the best four models. These findings suggest that the pulse period is the most crucial feature from the short time scale. For optimal prediction information on the short time scale (pulses [21], [22]) and information on the long time scale (chirps [11], [14]) are equally important. Also, in a taxonomic study [23] temporal features on both time scales (number of pulses per second, number of pulses per chirp, number of chirps per minute) were relevant for relating phylogeny to the species-specific song patterns.

### Logical AND-operation of the Time Scales

Calling songs of crickets carry information on short and long time scales, somewhat in analogy to words and phrases of human speech. How does the female cricket fuse auditory information that is present on the two distinct time scales? The response profile (Figure 3B) for different combinations of pulse and chirp periods showed a synergistic effect, that is only attractive pulse structures combined with attractive chirp structures drove highest phonotactic scores. This provided evidence for a logical AND-operation of the time scales and was in line with results from Grobe et al. [11] who interpolated behavioral measurements in the plane spanned by chirps per second and pulses per second, that is in the frequency domain. Notably, the combination of attractive pulse periods between 35 and 45 ms and unattractive chirp periods (greater than 500 ms) already caused intermediate responses. This again underlined the importance of the pulse period which we determined as the behaviorally most important feature of the short time scale.

The finding that the time scales are fused in an AND-operation can be interpreted with respect to the neuronal processing in the cricket brain. If our results had indicated a logical OR-operation of short and long time scales, then an independent, that is parallel processing of both time scales in the brain would have been likely. The result of the interdependence indicates that processing could be either parallel or serial. In the former case we expect from physiological experiments to find neuronal responses in the central brain that are independently tuned to either the short [24], [25] or the long time scale. The fusion of both information streams would happen only at a late stage of the brain network. Alternatively, in the latter case of serial processing we expect neural representations to be dependent on both time scales at an earlier stage of the brain network.

### Song Pattern Complexity in Crickets Versus Grasshoppers

Acoustic communication is also widely studied in grasshoppers. In mating behavior, male *Chorthippus biguttulus* grasshoppers produce courtship songs consisting of syllables that are grouped into phrases which in comparison with the songs of crickets exhibit a more complex song structure [26]–[28]. If females rate the song as attractive, they produce response signals that direct the male toward her [29]. Wittmann et al. [30] employed an approach similar to ours and analyzed courtship songs of the grasshopper *Chorthippus biguttulus* with artificial neural networks. Seven structural features of courtship songs were introduced and served as input to artificial neural networks. The linear correlation of 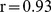 between the model’s predictions and the experimentally measured response probabilities was in a similar range as for our best 4-feature model. Wittmann et al. [30] also investigated the features that affect a female’s assessment of a male’s quality by excluding each song parameter once. In their case, none of the excluded features led to an increased performance of the corresponding reduced model. This indicates that the employed features are non-reducible and results in a feature space of at least seven dimensions. Thus, the processing of auditory information in female grasshoppers is more complex than in crickets.

### Non-linear Extension Improves Performance

A closer inspection of the best 4-feature model’s performance as shown in Figure 2B revealed a systematic mismatch between the behavioral measurements and the model prediction. For small experimental phonotactic scores (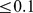) the model overestimated the attractiveness of the corresponding song patterns. Likewise, the model underestimated the attractiveness of models that were experimentally found to be highly attractive (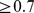). The same systematic bias was observed in the behavioral predictions by [30]. What could be the reason for this result and how could we improve model predictions? The classical artificial neural networks devised non-linear elements in the hidden layer while the output neuron computes a linear sum. We additionally applied a non-linear transformation of sigmoidal shape to the predictions of the model. In detail, we first used the training data set to choose 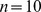 hidden neurons. Then, we chose the parameters 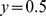 and 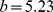 of the sigmoidal 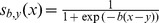 as they minimized the mean squared error between the experimentally measured phonotactic scores of the training data set and the sigmoidal transformed predictions. This improved the predictive power: on the test data set the error measure reduced to 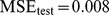 (as compared to 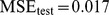) and the linear correlation coefficient was 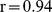. A possible interpretation of this result in a biological context involves two-step processing. In a first processing stage, the attractiveness of the stimulus pattern is evaluated. In a second stage, the outcome of this evaluation is non-linearly translated into behavior analog to a behavioral decision. To investigate this possibility it would be of interest to study in detail behavioral thresholds in individual animals [31].

### Towards Future Models of Neural Network Processing

We presented artificial neural networks that are suitable for predicting phonotactic scores of untested song patterns and thus for complementing behavioral as well as guiding electrophysiological studies. However, artificial neural networks do not attempt to model the natural neural processing of auditory information in the cricket brain. To improve our understanding of the underlying neuronal mechanisms during pattern recognition computational models of neural function are required that incorporate our anatomical, morphological, and physiological knowledge. Any such model should attempt to reproduce female phonotactic behavior and to provide testable hypotheses at the biophysical level.

Insects in general are well suited because they achieve the required tasks of pattern recognition and evaluation of the fitness parameters with relatively small brains. The cricket *Gryllus bimaculatus* is a well suited insect model for studying the neural basis of the processing of auditory information and the generation of choice behavior due to its highly limited neuronal resources. In the auditory pathway receptor neurons converge to two ascending neurons that project to a small number of neurons in the brain. Much is already known about the physiological properties [21], [32] of the ascending interneurons and ongoing work investigates the connectivity and physiological properties of the brain neurons. It has been shown that for varying pulse patterns some neurons match the average behavioral tuning [21], [24]. Several modeling approaches that use the cricket as a model system exist. Webb [33] investigates sound-seeking in crickets with robots. Mhatre and Balakrishnan [34] used a stochastic model to simulate the walking path of crickets. But, only few attempts have been made to model the neural mechanisms for pattern recognition in crickets. Benda and Hennig [35] showed that spike-frequency adaptation can generate intensity invariance in ascending neurons. In a preliminary study short term depression and short term facilitation in central brain synapses were suggested as plausible mechanisms for the parametric tuning on the short and long time scale [36]. Recently, based on their physiological investigation of central brain neurons, [24] suggested a network scheme that includes mutual excitation and inhibition of central brain neurons as a plausible alternative that awaits testing in a future neural network study.
